# WWP2 ameliorates oxidative stress and inflammation in atherosclerotic mice through regulation of PDCD4/HO-1 pathway: WWP2 interacted with PDCD4 ameliorates AS

**DOI:** 10.3724/abbs.2022091

**Published:** 2022-08-16

**Authors:** Xingye Wang, Lu Ma, Songlin Zhang, Qiang Song, Xumei He, Jun Wang

**Affiliations:** 1 Department of Structural Cardiology the First Affiliated Hospital of Xi’an Jiaotong University Xi’an 710061 China; 2 Department of Graduate School Xi’an Shiyou University Xi’an 710065 China

**Keywords:** atherosclerosis, WWP2, PDCD4, oxidative stress, ubiquitination

## Abstract

WWP2 is a HECT-type E3 ubiquitin ligase that regulates various physiological and pathological activities by binding to different substrates, but its role in atherosclerosis (AS) remains largely unknown. The objective of the present study is to investigate the role and underlying molecular mechanisms of WWP2 in endothelial injury. We found that WWP2 expression is significantly decreased in Apolipoprotein E (ApoE) ^–/–^ mice. Overexpression of WWP2 attenuates oxidative stress and inflammation in AS mice, while knockdown of *WWP2* has opposite effects. WWP2 overexpression alleviates oxidized low-density lipoprotein (ox-LDL)-induced human umbilical vein endothelial cell (HUVEC) injury, evidenced by the decreased oxidative stress levels and the secretion of inflammatory cytokines. Programmed cell death 4 (PDCD4) is identified as a potential substrate of WWP2. Co-immunoprecipitation (Co-IP) further demonstrates that WWP2 interacts with PDCD4, which is enhanced by ox-LDL treatment. Furthermore, the level of PDCD4 ubiquitination is significantly increased by WWP2 overexpression under the condition of MG132 treatment, while *WWP2* knockdown shows opposite results. Subsequently, rescue experiments demonstrate that *WWP2* knockdown further aggravates oxidative stress and inflammation in ox-LDL-treated HUVECs, while knockdown of *PDCD4* alleviates this effect. Moreover, the use of sn-protoporphyrin (SnPP), an inhibitor of HO-1 pathway, confirms that PDCD4 enhances endothelial injury induced by ox-LDL through inhibiting HO-1 pathway. In conclusion, our results suggest that WWP2 protects against atherosclerosis progression via the PDCD4/HO-1 pathway, which may provide a novel treatment strategy for atherosclerosis.

## Introduction

Atherosclerosis (AS) is a chronic vascular disease, which can result in various diseases such as coronary artery disease, stroke, and cerebrovascular disease [1]. The progression of AS involves many pathological events, such as the migration and proliferation of endothelial cells (ECs) and vascular smooth muscle cells (VSMCs), lipid deposition and persistent inflammation, as well as oxidative stress [2]. A strong relationship has been identified between endothelial function and the risk of cardiovascular diseases, especially AS. Meanwhile, emerging evidence supports that endothelial dysfunction or endothelial injury is an early marker of AS [3]. Therefore, it is urgent to explore the molecular pathogenesis of ECs in AS, which may provide effective treatment strategies for improving endothelial dysfunction to prevent AS.

Programmed cell death 4 (PDCD4) is an important tumor suppressor through inhibiting protein initiation complex formation [4]. A number of studies have demonstrated that PDCD4 is downregulated in various tumors, including ovarian cancer [5], breast cancer [6] and renal cell carcinoma [7]. In addition to the role of PDCD4 in tumor progression, PDCD4 was also shown to be involved in glucose and lipid metabolism disorders [8], oxidative stress [9], inflammatory responses [10], and intestinal microflora imbalance [11]. PDCD4 also plays a role in regulating cardiovascular diseases by inhibiting proliferation and inducing apoptosis of most cardiovascular cells, including VSMCs [12], cardiac myocytes [13], and fibroblasts [14]. PDCD4 expression is suppressed by cardiac progenitor cell-derived exosomal miR-21, which protects myocardial cells against oxidative stress-related apoptosis [15]. Upregulation of PDCD4 protects H _2_O _2_-induced brain microvascular endothelial cells against oxidative injury [16]. However, it was also demonstrated that deficiency of PDCD4 alleviates AS by inhibition of inflammatory pathways [17]. The reduction of PDCD4 expression in endothelial cells is associated with reduced atherosclerotic plaque area [18], and the inhibition of PDCD4 expression suppresses apoptosis and promotes proliferation of angiotensin II (AngII)-treated endothelial cell [19]. However, the potential molecular mechanism of PDCD4 in endothelial cells remains largely unknown.

Abnormal expression of PDCD4 contributes to disease progression via the ubiquitination-mediated degradation of PDCD4 under various conditions [20]. It was reported that the ubiquitin-proteasome–mediated degradation of PDCD4 is involved in AS; low level of PDCD4 is associated with reduced atherosclerotic plaque area [18]. Ubiquitination is a multi-step post-translational modification process of proteins.

WWP2, a member of the NEDD4 family of E3 ubiquitin ligases, is involved in many biological processes such as cell differentiation, cell cycle, immune response and apoptosis through regulating the target protein by ubiquitylation-dependent degradation [21]. WWP2 has protective effect against acute kidney injury by mediating p53 ubiquitylation [22]. WWP2 protects cartilage against osteoarthritis by Runt-related transcription factor 2 (Runx2) poly-ubiquitination and degradation to suppress Adamts5 expression [23]. It has been reported that knockout of *WWP2* specifically in myocardium decreases the level of PARP1 ubiquitination, thus aggravating ISO-induced myocardial hypertrophy, heart failure, and myocardial fibrosis [24]. WWP2 modulates hypertensive angiopathy by regulating SIRT1-STAT3, and WWP2 suppression in VSMCs alleviates hypertensive angiopathy *in vitro* and *in vivo* [25]. Interestingly, recent findings revealed that knockout of *WWP2* reduced the ubiquitination and degradation of endothelial injury factor Septin4, thus aggravating angiotensin II/oxidative stress-induced endothelial injury and vascular remodeling after endothelial injury [26]. Moreover, endothelial injury is the initial event and major cause of multiple cardiovascular diseases such as AS and hypertensive vascular diseases [ 27, 28] . Thus, we hypothesized that WWP2 may play an important role in AS through protecting HUVECs from endothelial injury.

In this study, PDCD4 was found to be significantly upregulated in human umbilical vein endothelial cells (HUVECs) treated with ox-LDL. Mechanistically, we found that WWP2 is an interacting protein of PDCD4 by using bioinformatics online tools, and Co-IP results further verified the interaction between PDCD4 and WWP2. Our results suggested that WWP2 binds with and downregulated PDCD4 by ubiquitin degradation, thus reducing oxidative stress and inflammation of endothelial cells in atherosclerotic mice. This study provides novel mechanistic insights into the role of WWP2 in the progression of AS.

## Materials and Methods

### Ethics statement

All animal experiments were conducted under the approval of the Animal Ethics Committee of the First Affiliated Hospital of Xi’an Medical University (Xi’an, China) and in strict accordance with the Guide for the Care and Use of Laboratory Animals published by NIH (Bethesda, USA).

### Establishment of AS model in ApoE ^–/–^ mice

Male ApoE ^–/–^ mice based on C57/BL6 background (aged seven weeks old, and weighing 18–20 g) were purchased from Beijing HFK Bioscience Co., Ltd (Beijing, China) to establish mouse models of AS. Ten male C57/BL6 mice (Wild-type; aged seven weeks old, and weighing 18–20 g) were also purchased from Beijing HFK Bioscience Co., Ltd as a normal control group. The mice were housed at 22–23°C, 55%–60% humidity, 12 h light/ 12 h dark cycle, with free access to food and water. After one week of acclimatization, the mice were fed with a high-fat diet (21% fat and 0.15% cholesterol) for 10 weeks. ApoE ^–/–^ mice were randomly divided into 5 groups (12 in each group): ApoE ^–/–^, ApoE ^–/–^ + Vector, ApoE ^–/–^ + Ad-WWP2, ApoE ^–/–^ + NC shRNA, and ApoE ^–/–^ + WWP2 shRNA groups. WWP2 adenoviruses overexpressing vector (Ad-WWP2), WWP2 short hairpin RNA (shRNA), or their negative controls were injected into ApoE ^–/–^ mice through the tail vein once every two weeks. After that, the mice were euthanized, and the coronary artery tissue samples were collected.

### Cell culture and ox-LDL treatment

HUVECs, VSMCs, THP-1 monocytes, and HEK 293T cells were purchased from Shanghai Cell Bank of the Chinese Academy of Sciences (Shanghai, China). Cells were cultured in DMEM containing 10% fetal bovine serum (FBS), 100 U/mL streptomycin, and 100 U/mL penicillin in a humidified atmosphere containing 5% CO _2_ at 37°C. The ox-LDL (Solarbio, Beijing, China) at a series of concentrations (0, 25, 50, and 100 μg/mL) was added into HUVECs and incubated for 24 h to induce cell injury. Cycloheximide, SnPP and MG132, which were purchased from Sigma (St Louis, USA), were also applied in the treatment of ox-LDL pre-treated HUVECs

### Cell transfection

Ad-WWP2, WWP2 shRNA and their negative controls were purchased from GenScript Biotech Corp. (Nanjing, China). Small interfering RNAs against PDCD4 and WWP2 (PDCD4 siRNA and WWP2 siRNA), and the negative control (Scramble) were purchased from Sangon Biotech Co., Ltd. (Shanghai, China). All these plasmids and oligonucleotides were transfected into HUVECs by using Lipofectamine ^®^ 3000 reagent (Thermo Fisher Scientific, Waltham, USA). The sequence information of all shRNAs, siRNAs and their negative controls used in this study were provided in Table 1. At 48 h after transfection, cells were harvested for further study.

**Table:** **Table 1** The sequences of shRNAs and siRNAs used in this study

shRNA/siRNA	Sequence (5′→3′)
NC shRNA	CACCGCGGATGGCAGTCTGGAATAACGAATTATTCCAGACTGCCATCCGC
WWP2 shRNA	CACCGGGGGAAAAAATTTCCCGGGTCGAACCCCCTTTTTTAAAGGGCCCA
Scrambled siRNA	AGCCGCTTAGGAATGCTCUUU
PDCD4 siRNA	GAACTGGAAGTACCTCATTT
WWP2 siRNA	AGGAGGTTCTGCCTGTAATTT

### Cell viability assay

MTT assay was used to detect the cell viability. Briefly, cells were seeded in 96-well plates at a density of 5×10 ^3^ cells/well and were subjected to different treatment. Then, MTT solution (20 μL, 5 mg/mL; Sigma, St Louis, USA) was added and incubated with cells at 37°C for 4 h. After removing the medium, 0.1 mL dimethyl sulfoxide (Sigma) was added to dissolve the formazan product for 15 min. Subsequently, the absorbance was measured at 490 nm with a microplate reader (BioTek, Winooski, USA).

### Cell apoptosis analysis

The apoptosis of HUVECs was evaluated by using Annexin V/FITC and propidium iodide (PI) apoptosis detection kit (Becton Dickinson, Pasadena, USA) according to the manufacturer’s instructions. Briefly, treated cells were collected, and were suspended in Annexin-binding buffer, followed by staining with Annexin V- FITC/PI for 15 min in the dark at room temperature. Subsequently, the stained cells were analyzed by flow cytometry using the CYTOMICS FC 500 flow cytometer (Beckman Coulter, Brea, USA).

### Enzyme linked immunosorbent assay (ELISA)

The contents of the cytokines IL-6, IL-1β and TNF-α in mouse serum and the cell supernatant were detected with Mouse/Human IL-6, IL-1β and TNF-α SimpleStep ELISA Kits (Abcam, Cambridge, USA), respectively.

### Oxidative stress measurement

For the detection of oxidative stress in atherosclerotic mice, blood samples were harvested from the sacrificed mice, and serum was collected after centrifugation at 2000 *g* for 10 min at 4°C. Then, serum levels of malondialdehyde (MDA), superoxide dismutase (SOD), glutathione (GSH) and glutathione peroxidase (GSH-Px) were determined using corresponding enzyme-linked immunosorbent assay (ELISA) kits (Nanjing Jiancheng Biological Engineering Institute, Nanjing, China). For the detection of oxidative stress in cells with indicated treatment, cells were collected after centrifugation at 1000 *g* for 10 min at 4°C, and ROS, MDA and SOD levels were also determined using ELISA kits.

### eNOS activity detection

Serum levels of total cholesterol (TG), triglyceride (TC), high-density lipoprotein (HDL) and low-density lipoprotein (LDL) were measured using commercial biochemical kits (Nanjing Jiancheng Bioengineering Institute). For the determination of nitric oxide production and eNOS activity in the media of HUVECs, the level of nitric oxide was quantified using an assay kit (Nanjing Jiancheng Biological Engineering Institute, Nanjing, China) according to the manufacturer’s protocol, and the eNOS activity was determined using an ELISA kit (Nanjing Jiancheng Biological Engineering).

### Quantitative real-time polymerase chain reaction(RT-qPCR)

Total RNA was extracted from the coronary artery tissues and cells using Trizol reagent (Life Technologies, Carlsbad, USA) following the manufacturer’s instructions and then reverse-transcribed to cDNA using PrimeScript RT reagent kit (Thermo Fisher Scientific). The quantitative PCR was performed by using the SYBR ® Premix Ex Taq™ reagent (BioTeke, Beijing, China) on the CFX96 qPCR machine (Invitrogen, Carlsbad, USA) with the following steps: 10 min at 95°C; 35 cycles of 15 s at 95°C, 20 s at 60°C and 15 s at 72°C. The levels of mRNAs were calculated by the 2 ^−ΔΔCt^ method, and normalized to that of *GAPDH* as an internal reference. The primer sequences used in this study are listed in the Table 2.

**Table:** **Table 2** Sequences of primers used in RT-qPCR

Gene	Sequence (5′→3′)
*PDCD4*	F: AAGAAAGGTGGTGGCAGGAGG
	R: TGACTAGCCTTCCCCTCCAA
*HO-1*	F: CGACAGCATGTCCCAGGATT
	R: TCGCTCTATCTCCTCTTCCAGG
*VCAM-1*	F: GATACAACCGTCTTGGTCAGCCC
	R: ATTGCCACAAGCAGAAAGACA
*ICAM-1*	F: AAACGGGAGATGAATGGT
	R: TCTGGCGGTAATAGGTGTA
*p47*	F: TCCCAAGTGGTTTGACGG
	R: CCTCCTCTTTCTGGCTGTG
*WWP2*	F: GAGATGGACAACGAGAAG
	R: CTCCTCAATGGCATACAG
*GAPDH*	F: GGTCGGGCAGGAAAGAGGGC
	R: CTAATCTTCTCTGTATCGTTCC

### Western blot analysis

Proteins from cells and the coronary artery tissues were extracted using radioimmunoprecipitation assay (RIPA) lysis buffer (Beyotime, Beijing, China). The protein was quantified by BCA protein assay, and 25 μg proteins were separated by SDS-PAGE and transferred to PVDF membranes (Millipore, Billerica, USA). After being blocked with 5% non-fat milk for 1 h, the membranes were incubated with the primary antibodies overnight at 4°C. The primary antibodies used in this study were as following: anti-WWP2 (1:5000; ab103527, Abcam), anti-ICAM-1 (1:1000; #4915, Cell Signaling Technology, Beverly, USA), anti-VCAM-1 (1:1000; #13662, Cell Signaling Technology), anti-PDCD4 (1:5000; ab51495, Abcam), anti-HO-1 (1:2000; ab13243, Abcam), anti-p47 (1:1000; #4312, Cell Signaling Technology), anti-HA (1:1000; 3724S, Cell Signaling Technology), anti-Myc (1:1000; 2276S, Cell Signaling Technology), anti-Flag (1:1000; 14793S, Cell Signaling Technology) and GAPDH (1:5000; ab8245, Abcam). Next, the membranes were incubated with HRP-labeled secondary antibodies at room temperature for 2 h. Then, the protein blots were visualized by using ECL kit (Thermo Fisher Scientific), and the gray level of bands were analyzed by using Image J software (NIH, Bethesda, USA). The relative protein levels were normalized to GAPDH.

### Co-immunoprecipitation assay

Cells were washed twice with protease inhibitor and solubilzed with a marker solution buffer. The lysates were incubated with antibodies (antibody/cell lysate= 1 μg/mg) for 2–3 h, and then incubated with 30 μL of Protein A/G immunoprecipitate (B23202; Biotool, Kirchberg, Switzerland) for 12 h at 4°C. After thorough mixing and washing, the immune complex was detected by SDS-PAGE. Then, the samples underwent successive incubations with primary antibodies at 4°C overnight, followed by incubation with the secondary antibodies for 1 h at room temperature. GAPDH was used for normalization, and Image J software (NIH) was employed to quantify the immunoreactive bands.

### Ubiquitination assay

HA-ubiquitin and Myc-PDCD4 were co-transfected into HEK 293T cells in the absence of Flag-WWP2 or WWP2 siRNA or their negative controls for 48 h. Then, cells were lysed with 1% SDS buffer (Tris, pH 7.5, 0.5 mM EDTA, and 1 mM DTT) and boiled for 10 min. The cell lysates were incubated with anti-PDCD4 antibody (antibody/cell lysate= 1 μg/mg) and 30 μL of protein A/G immunoprecipitation magnetic beads or 30 μl of anti-Myc magnetic beads for 12 h at 4°C. Analyses of PDCD4 ubiquitination were performed by immunoblotting using anti-HA antibodies (Abcam).

### Immunofluorescence analysis

Cellular immunofluoresence was used following the well-established procedure. The primary aortic endothelial cells were isolated and cultured as previously described [29]. Briefly, cells in different groups were fixed with 4% paraformaldehyde, followed by permeabilization with 0.5% Triton X-100 in PBS for 20 min. After being blocked with 1% BSA in PBS for 30 min, cells were incubated with anti-WWP2 (1:250; ab207298; Abcam) or PDCD4 (1:500; ab79405; Abcam) antibody overnight at 4°C. Subsequently, cells were probed with florescence-labeled secondary antibody (Abcam). The nuclei were stained with DAPI for 3 min under dark condition. Finally, a fluorescence microscope (Eclipse 90i; Nikon, Tyoko, Japan) was used to visualize and capture images.

### Statistical analysis

All data are expressed as the mean±standard error of the mean (SEM) of at least three independent experiments. Statistical differences between groups were analyzed with one-way analysis of variance (ANOVA) or Student’s *t*-test, which were performed using SPSS 22.0. *P*<0.05 was considered statistically significant.

## Results

### WWP2 overexpression ameliorates AS development in ApoE ^–/–^ mice

**Figure 1:**
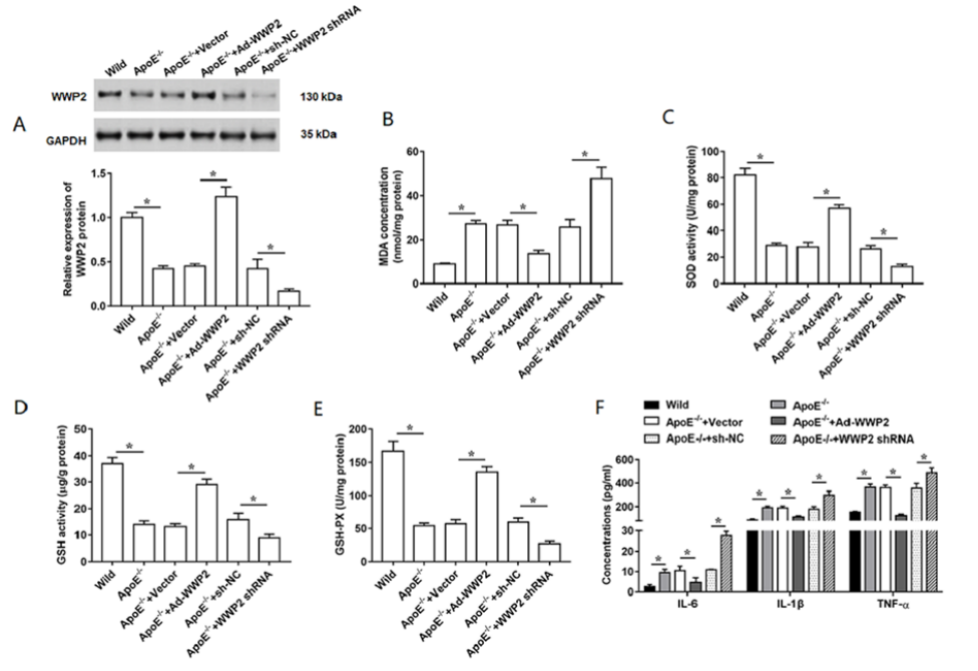
Figure 1 Effects of WWP2 overexpression on AS development The AS model (ApoE –/– with high-fat diet) was established, and Ad-WWP2, WWP2 shRNA and their respectively controls were injected via tail vein (12 mice/group). (A) Relative expression level of WWP2 was measured in ApoE –/– mice. (B–E) Levels of MDA, SOD, GSH and GSH-Px were detected by using ELISA kits. (F) Concentrations of inflammatory cytokines, including IL-6, IL-1β and TNF-α in serum were measured using ELISA kits. Data are expressed as the mean±SEM. Student’s t-test was used for the comparison in this study. * P<0.05.

The atherosclerotic mice model was established in ApoE ^–/–^ mice with high-fat diet for 10 weeks, and Ad-WWP2 and WWP2 shRNA were injected via tail vein once every two weeks. We observed that WWP2 expression was significantly decreased in coronary artery tissues from AS mice compared with the wild-type mice, which was reversed by the injection of Ad-WWP2 ( Figure 1A). Moreover, the level of oxidative stress was elevated in AS mice, while overexpression of WWP2 showed lower oxidative stress level, which was evidenced by the determination of MDA concentration, and the activities of SOD, GSH and GSH-PX ( Figure 1B–E). Then, we detected the concentrations of several inflammatory cytokines in the serum of ApoE ^–/–^ mice, and the results indicated that the secretion of IL-6, IL-1β and TNF-α showed a remarkably reduction in WWP2-overexpressing ApoE ^–/–^ mice ( Figure 1F). Additionally, serum lipid levels were determined. As shown in Table 3, serum TG, TC, and LDL levels displayed obvious increases in ApoE ^–/–^ mice compared with those in wild-type mice. In contrast, serum HDL level was decreased in ApoE ^–/–^ mice compared with that in wild-type mice. However, injection of Ad-WWP2 markedly reversed the serum TG, TC, LDL and HDL levels in ApoE ^–/–^ mice. Moreover, we also performed some experiments to assess the role of *WWP2* knockdown in AS model mice. The results revealed that knockdown of *WWP2* could significantly increase the levels of oxidative stress and inflammatory cytokines as well as serum lipid in ApoE ^–/–^ mice fed with high-fat diet ( Figure 1A–F and Table 3).

**Table:** **Table 3** Effect of WWP2 overexpression on serum lipid levels

Group	TG (mg/dL)	TC (mg/dL)	HDL (mg/dL)	LDL (mg/dL)
Wild	25.1±8.5	142.3±18.4	98.4±15.2	16.3±8.5
ApoE ^–/–^	188.6±15.3*	683.8±38.5*	26.8±10.5*	189.6±27.6*
ApoE ^–/–^+Vector	191.4±18.9*	678.9±41.2*	24.5±8.8*	192.5±21.3*
ApoE ^–/–^+Ad-WWP2	68.3±5.4 ^#^	256.3±35.1 ^#^	82.7±4.6 ^#^	57.3±18.4 ^#^
ApoE ^–/–^+NC shRNA	183.5±13.1*	674.1±57.9*	23.7±3.5*	207.5±19.9*
ApoE ^–/–^+WWP2 shRNA	317.6±34.7 ^&^	835.5±65.5 ^&^	11.4±2.0 ^&^	341.5±41.5 ^&^

TG: total cholesterol; TC: triglyceride; HDL: high-density lipoprotein; LDL: low-density lipoprotein. * *P*<0.05 vs Wild group; ^#^ *P*<0.05 vs ApoE ^–/–^+Vector group; ^&^ *P*<0.05 vs ApoE ^–/–^+NC-shRNA group.

### WWP2 is downregulated in ox-LDL-induced HUVECs

**Figure 2:**
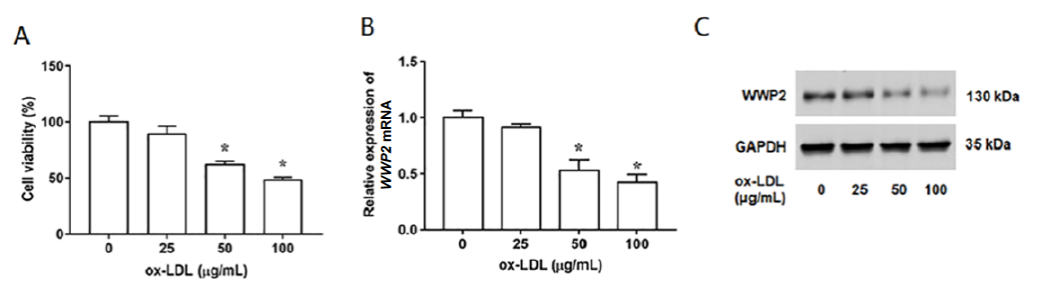
Figure 2 Expression level of WWP2 in ox-LDL-induced HUVECs The HUVECs were treated with ox-LDL at a series of concentrations (0, 25, 50, or 100 μg/mL) for 24 h. (A) Cell viability was detected by MTT assay. (B) Expression level of WWP2 mRNA was measured by RT-qPCR. (C) Expression level of WWP2 protein was measured by western blot analysis. n=5. Data are expressed as the mean±SEM. Student’s t-test was used for the comparison in this study. * P<0.05 vs 0 μg/mL group.

In this study, ox-LDL was added into HUVECs and incubated for 24 h to induce endothelial damage. HUVECs were treated with ox-LDL at a series of concentrations (0, 25, 50, or 100 μg/mL) for 24 h, and the cell viability was gradually decreased with the increase of ox-LDL concentrations ( Figure 2A). Moreover, we detected the WWP2 expression in ox-LDL-stimulated HUVECs, and the results indicated the expression of WWP2 were significantly reduced in a dose-dependent manner at both mRNA and protein levels ( Figure 2B,C). Thus, ox-LDL at a concentration of 100 μg/mL was used in the subsequent experiments. Furthermore, the primary aortic endothelial cells were applied to evaluate the level and the localization of WWP2 by immunofluorescence microscopy. The results showed that the expression level of WWP2 was decreased in ox-LDL-stimulated-aortic endothelial cells ( Supplementary Figure S1A). We also detected the expression of WWP2 in ox-LDL-stimulated VSMCs and THP-1 macrophages, and the results showed that the expression level of WWP2 was significantly decreased in ox-LDL-stimulated VSMCs ( Supplementary Figure S2A), as well as in ox-LDL-induced THP-1 macrophages ( Supplementary Figure S2B).

### Overexpression of WWP2 reduces oxidative stress and inflammation in ox-LDL-induced HUVECs

**Figure 3:**
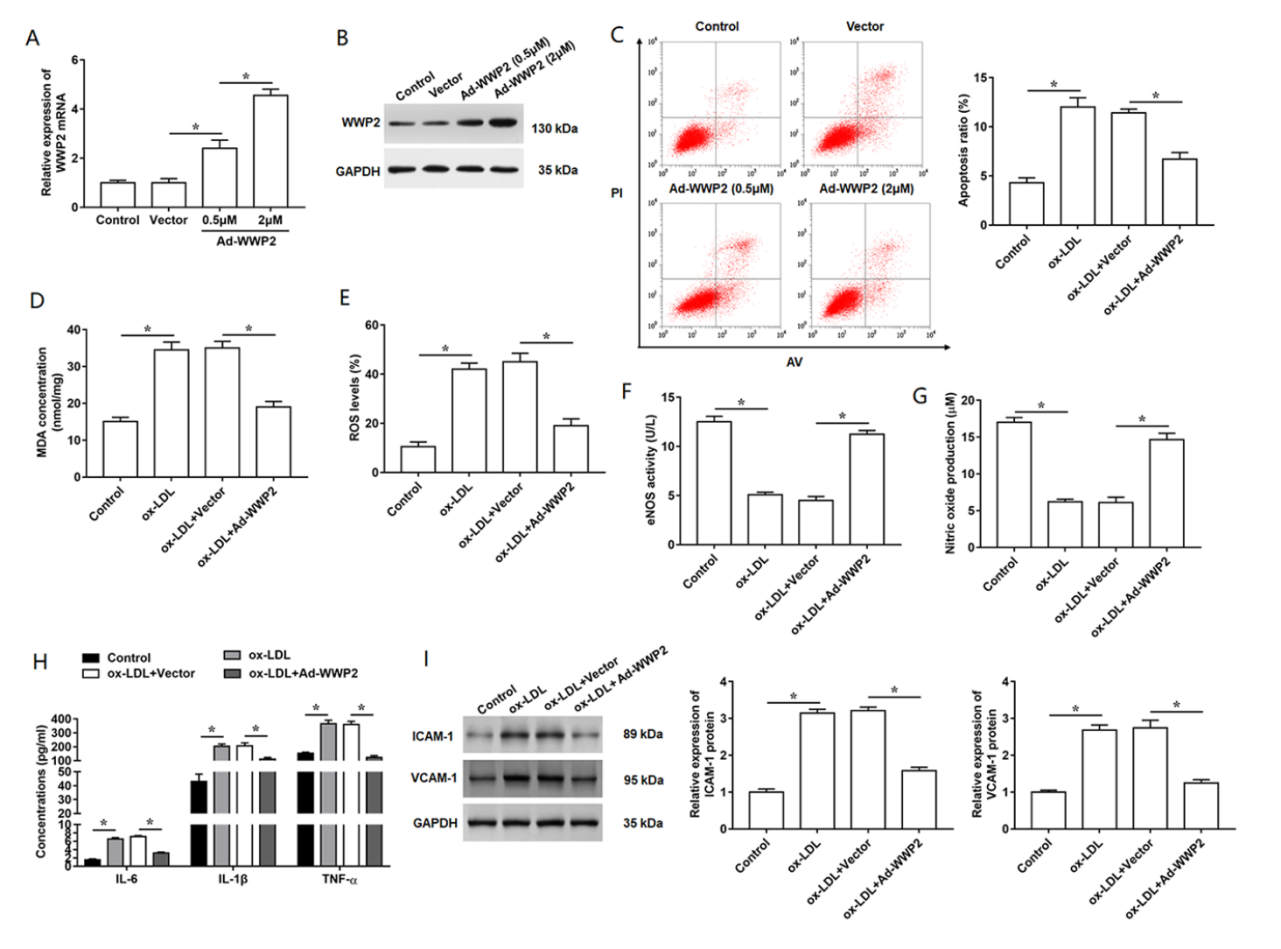
Figure 3 Overexpression of WWP2 reduces oxidative stress and inflammatory response in ox-LDL-treated HUVECs (A,B) Transfection efficiencies of WWP2 overexpression in HUVECs transfected with Ad-WWP2 at the concentration of 0.5 μM or 2 μM. After transfection of 2 μM Ad-WWP2 for 48 h, cells were incubated with 100 μg/mL ox-LDL. (C) Apoptosis ratio in HUVECs subjected to different treatment was analyzed by flow cytometry. (D,E) Levels of MDA and ROS were detected by using ELISA kits. (F) The content of eNOS in HUVECs were detected using ELISA kit. (G) Nitric oxide release in the cultured medium of HUVECs was measured by using the assay kit. (H) Concentrations of inflammatory cytokines in the culture supernatant were measured by using ELISA kits. (I) The expression levels of VCAM-1 and ICAM-1 proteins were detected by western blot analysis. n=5. Data are expressed as the mean±SEM. Student’s t-test was used for the comparison in this study. * P<0.05.

To explore the effect of WWP2 on ox-LDL-induced endothelial cell damage, HUVECs were transfected with Ad-WWP2, followed by treatment with 100 μg/mL ox-LDL. First, we detected the transfection efficiency of Ad-WWP2 in HUVECs ( Figure 3A,B). Then, the results showed that the apoptosis ratio was promoted in ox-LDL-treated HUVECs, while overexpression of WWP2 reversed it ( Figure 3C). Treatment with ox-LDL induced cell oxidative stress, evidenced by the increase of MDA and ROS levels, which was reversed by the transfection with Ad-WWP2 ( Figure 3D,E). Moreover, we observed the markers of endothelial cell damage, including eNOS and NO in ox-LDL-treated HUVECs, and the results showed that the effects of ox-LDL treatment on eNOS and NO levels were weakened by WWP2 overexpression ( Figure 3F,G). The increased secretion of inflammatory cytokines, including IL-6, IL-1β and TNF-α induced by ox-LDL treatment was obviously reduced in WWP2-overexpressing HUVECs ( Figure 3H). Subsequently, we found that the promoting effects of ox-LDL treatment on the expression of adhesion-related molecule, including ICAM-1 and VCAM-1, were partially abolished by the overexpression of WWP2 ( Figure 3I). Collectively, these data suggested that overexpression of WWP2 could improve endothelial cell damage by reducing the oxidative stress and inflammation in ox-LDL-induced HUVECs.

### WWP2 binds directly with PDCD4, and degrades PDCD4 through the ubiquitin pathway

**Figure 4:**
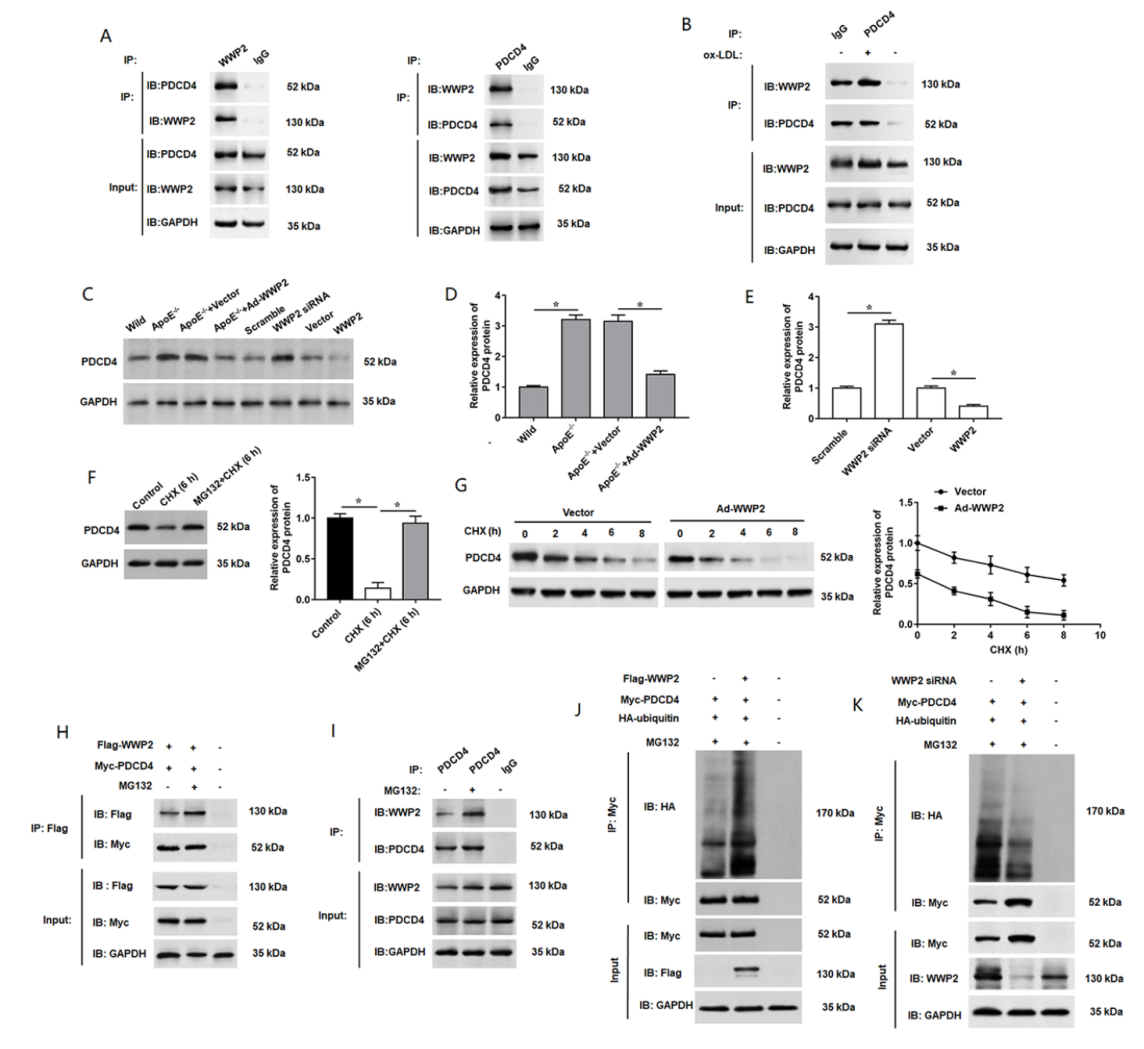
Figure 4 PDCD4 is ubiquitinated by WWP2 through a protein-protein interaction stimulating degradation (A) Co-IP assay of PDCD4 interaction with WWP2. Cell extracts were incubated with anti-WWP2 antibody (left panel) or anti-PDCD4 antibody (right panel), and the precipitated complexes were further blotted using antibodies against PDCD4 or WWP2. Whole cell lysates served as positive control for western blot analysis. IgG served as negative control for immunoprecipitation. (B) Endogenous interaction between PDCD4 and WWP2 was enhanced by treatment with 100 μg/mL ox-LDL for 24 h, which was evaluated by co-immunoprecipitation using anti-PDCD4 antibody. GAPDH was used as a loading control. (C,D) The expression level of PDCD4 protein was measured in ApoE –/– mice. (C,E) Expression of PDCD4 protein in HUVECs transfected with WWP2 siRNA or WWP2 overexpression vector. (F) HEK 293T cells were treated with 50 mg/mL cycloheximide alone or together with 10 mM MG132 for 6 h. The expression level of PDCD4 was detected. (G) Expression of PDCD4 was assessed at different durations of CHX administration with or without WWP2 overexpression in 293T cells. Co-immunoprecipitation was performed to determine the interaction between endogenous (H) and exogenous (I) WWP2 and PDCD4 with or without MG132 treatment in HEK 293T cells. (J) Flag-WWP2, Myc-PDCD4, and HA-Ub were coexpressed with or without MG132 treatment. PDCD4 was isolated by IP, and PDCD4 ubiquitination levels were assessed with anti-HA antibodies. (K) WWP2 siRNA, Myc-PDCD4 and HA-Ub were coexpressed and applied with or without MG132. PDCD4 was isolated by IP, and PDCD4 ubiquitination levels were assessed with anti-HA antibodies. n=5. Data are expressed as the mean±SEM. One-way ANOVA was used for the comparison in this study. * P<0.05.

The above results demonstrated the positive effect of WWP2 on ox-LDL-induced endothelial damage. However, the mechanism is still not clear. We identified that PDCD4 is a WWP2-interacting protein by Co-IP assay. The results indicated that immunoprecipitates of sample proteins using antibodies against WWP2 (IP-WWP2) was positive for PDCD4 ( Figure 4A, left panel), and using antibodies against PDCD4 (IP-PDCD4) was positive for WWP2 ( Figure 4A, right panel). Additionally, we treated HUVECs with or without ox-LDL, and then performed immunoprecipitation assay. The results revealed that the interaction was enhanced by ox-LDL treatment ( Figure 4B). Subsequently, we found the PDCD4 expression was increased in ApoE ^–/–^ mice, while injection of Ad-WWP2 markedly reversed this promoting effect ( Figure 4C,D). Then, we verified the effect of WWP2 on the expression of PDCD4 by transfection of HUVECs with WWP2 siRNA or Ad-WWP2, and the results indicated that PDCD4 was upregulated by knockdown of *WWP2*, and downregulated by overexpression of WWP2 ( Figure 4C,E). Moreover, we evaluated the level and the localization PDCD4 in the primary aortic endothelial cells by immunofluorescence microscopy. The results showed that the expression level of PDCD4 was increased in ox-LDL-stimulated-aortic endothelial cells ( Supplementary Figure S1B). Consistently, compared with the control group, the expression level of PDCD4 was significantly upregulated in ox-LDL-stimulated VSMCs ( Supplementary figure S2A), as well as in ox-LDL-induced THP-1 macrophages ( Supplementary figure S2B).

As we all know, WWP2 is a HECT-type E3 ubiquitin ligase. To investigate whether WWP2 decreases the expression of PDCD4 by inhibiting PDCD4 transcription or promoting PDCD4 proteasome degradation, the protein synthesis inhibitor cycloheximide (CHX) and proteasome inhibitor MG132 were added to evaluate the effects of WWP2 on PDCD4 in 293T cells. PDCD4 protein level was significantly decreased in the presence of CHX (5 μM), while MG132 (100 nM) treatment recovered PDCD4 protein level ( Figure 4F), which suggested that ubiquitin system plays an important role in PDCD4 degradation. Subsequently, WWP2-overexpressing HEK 293T cells were treated with CHX at certain intervals to evaluate the impact of WWP2 on the stability of PDCD4. The data indicated that under the conditions that protein synthesis was inhibited, overexpression of WWP2 dramatically reduced PDCD4 level, and PDCD4 expression was decreased with the increase in time of CHX treatment, indicating that WWP2 accelerates the degradation rate of PDCD4 and has an inhibiting effect on the stability of PDCD4 ( Figure 4G). Thus, to confirm whether WWP2 affects the level of PDCD4 ubiquitination, we performed coimmunoprecipitation to determine the ubiquitination effect of WWP2 on PDCD4 in 293T cells. Our data suggested that the MG132 treatment remarkedly enhanced the interaction between exogenous WWP2 and PDCD4 ( Figure 4H) as well as the interaction between endogenous WWP2 and PDCD4 ( Figure 4I). Consistently, the level of PDCD4 ubiquitination was significantly increased by overexpression of WWP2 under the condition of MG132 treatment ( Figure 4J), while knockdown of *WWP2* showed opposite results ( Figure 4K). These findings suggested that WWP2 binds directly with PDCD4 and mediates PDCD4 degradation through the ubiquitin proteasome pathway.

### Knockdown of PDCD4 weakens the effects of *WWP2* knockdown on ox-LDL- induced oxidative stress and inflammation

**Figure 5:**
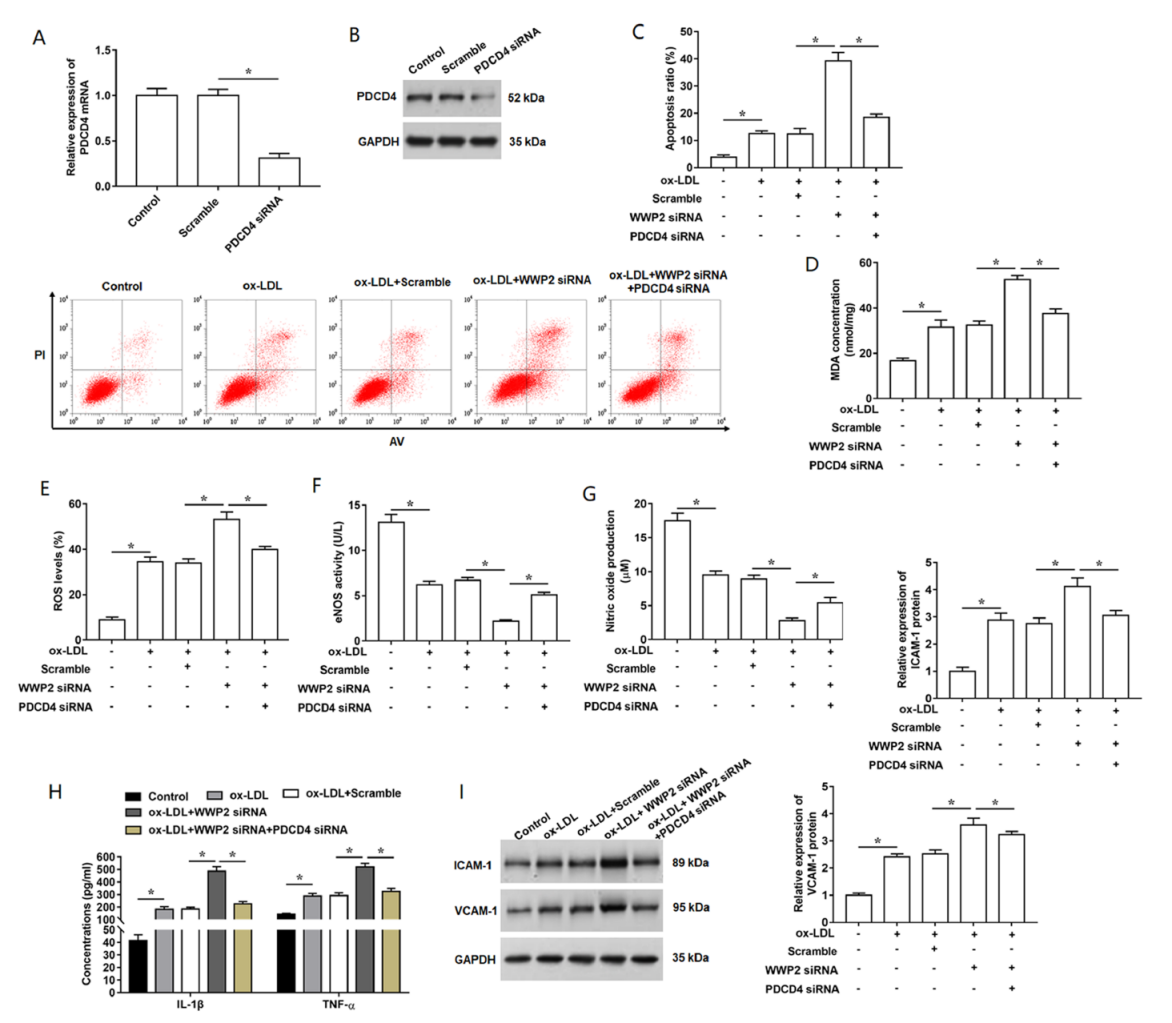
Figure 5 **Silencing of** ***PDCD4*** **rescues the effects of WWP2 knockdown on ox-LDL-induced oxidative stress and inflammatory response in HUVECs** (A,B) Transfection efficiency of PDCD4 siRNA in HUVECs at mRNA and protein levels was detected. Then, after transfection with WWP2 siRNA alone or together PDCD4 siRNA, cells were treated with ox-LDL. (C) Apoptosis ratio in HUVECs subjected to different treatment was analyzed by flow cytometry. (D,E) Levels of MDA and ROS levels were detected. (F) The content of eNOS in HUVECs were detected using ELISA kit. (G) Nitric oxide release in the cultured medium of HUVECs was measured by using the assay kit. (H) Concentrations of IL-1β and TNF-α in the culture supernatant were measured by using ELISA kits. (I) The expression levels of VCAM-1 and ICAM-1 proteins were detected by western blot analysis. n=5. Data are expressed as the mean±SEM. Student’s t-test and One-way ANOVA were used for the comparison in this study. * P<0.05.

Then, we performed a series of rescue experiments to further explore the role of WWP2 and PDCD4 in AS. PDCD4 siRNA was transfected into HUVECs, and PDCD4 expression was detected by RT-qPCR and western blot analysis ( Figure 5A,B). Apoptosis ratio in HUVECs subjected to different treatment was analyzed by flow cytometry, and it was found that knockdown of *WWP2* promoted ox-LDL-induced cell apoptosis, which was reversed by the treatment with PDCD siRNA ( Figure 5C). Oxidative stress levels induced by WWP2 siRNA were reduced in PDCD4-silenced HUVECs, which was evidenced by the decrease of MDA and ROS levels ( Figure 5D,E). Moreover, the inhibitory effects of WWP2 siRNA treatment on eNOS and NO levels were weakened by the knockdown of *PDCD4* in ox-LDL-treated HUVECs ( Figure 5F,G). The increased secretion of inflammatory cytokines, including IL-1β and TNF-α induced by WWP2 siRNA treatment was obviously reduced by PDCD4 silencing ( Figure 5H). Additionally, the expression levels of ICAM-1 and VCAM-1 were significantly increased by the transfection with WWP2 siRNA, which was effectively reversed by knockdown of PDCD4 ( Figure 5I). Taken together, these results verified that downregulation of PDCD4 attenuates the promoting effects of WWP2 knockdown on endothelial cell damage.

### PDCD4 promotes ox-LDL-induced endothelial injury by inhibiting the HO-1 pathway

**Figure 6:**
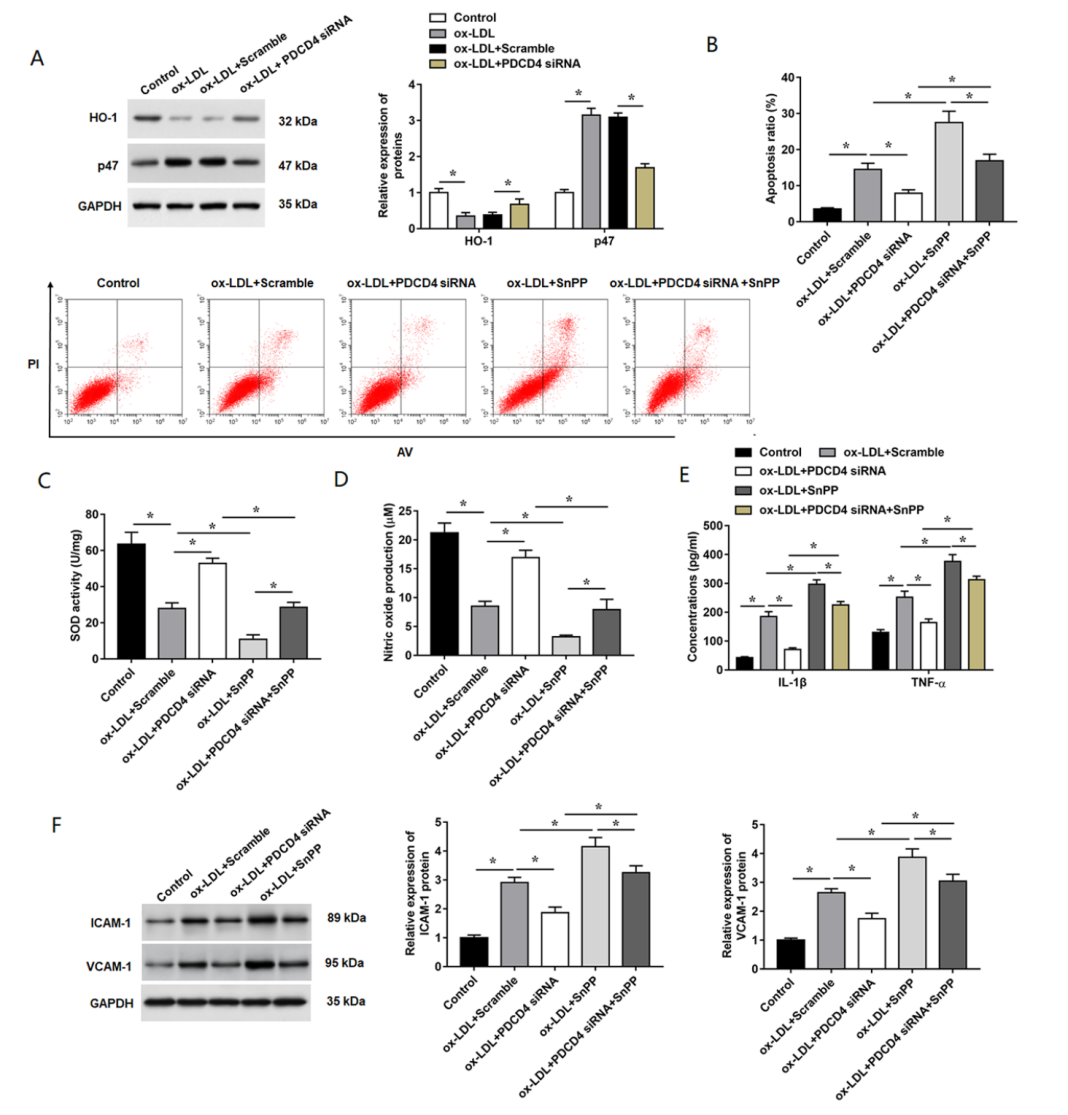
Figure 6 PDCD4 promotes ox-LDL-induced HUVECs injury through inhibiting the HO-1 pathway (A) Relative expressions of HO-1 and p47 protein were detected by western blot analysis in HUVECs subjected to PDCD4 siRNA and ox-LDL treatment. HUVECs were pre-treated with HO-1 inhibitor, SnPP (20 μM), for 2 h, followed by the PDCD siRNA transfection. Then, the cells were exposed to ox-LDL for an additional 24 h. (B) Apoptosis ratio in HUVECs subjected to different treatment was analyzed by flow cytometry. (C) SOD activity was detected. (D) Nitric oxide release in the cultured medium of HUVECs was measured by using the assay kit. (E) Concentrations of IL-1β and TNF-α in the culture supernatant were measured by using ELISA kits. (F) The expression levels of ICAM-1 and VCAM-1 proteins were detected by western blot analysis. n=5. Data are expressed as the mean±SEM. Student’s t-test and One-way ANOVA were used for the comparison in this study. * P<0.05.

HO-1 is an antioxidant stress response protein which plays an important role in protecting against oxidative stress [30]. p47 is a subunit of NADPH-dependent oxidoreductase, in which NADPH plays an important role in the development by acting as a precursor of ROS [31]. The results showed that HO-1 expression was suppressed and p47 expression was promoted, while knockdown of PDCD4 effectively reversed these effects ( Figure 6A). To further reveal the role of HO-1 in PDCD4-regulated AS, HO-1 inhibitor, SnPP, was used to treat HUVECs. As shown in Figure 6B–F, the administration of SnPP (250 nM) significantly increased ox-LDL-induced cell apoptosis ratio, oxidative stress level and release of inflammatory cytokines in HUVECs. Figure 6B indicated that PDCD4 siRNA significantly inhibited cell apoptosis induced by xo-LDL, while SnPP pre-treatment showed opposite results ( Figure 6B). Moreover, SnPP pre-treatment partially abolished *PDCD4* knockdown-induced decrease of SOD activity in ox-LDL-treated cells ( Figure 6C). The NO level was decreased by the knockdown of *PDCD4* in ox-LDL-treated HUVECs, but the application of SnPP weakened this effect ( Figure 6D). The increased secretion of inflammatory cytokines and expression of adhesion-related molecule were significantly decreased by the transfection with PDCD4 siRNA in ox-LDL-stimulated HUVECs, which was effectively reversed by SnPP treatment ( Figure 6E,F). Furthermore, we detected the expression of HO-1 and p47 at the protein and mRNA levels in the coronary artery tissues of AS mice by western blot analysis and RT-qPCR. The results showed that overexpression of WWP2 significantly promoted the expression levels of HO-1 protein and mRNA in AS model mice, while *WWP2* knockdown showed opposite results ( Supplementary Figures S3 and S4). And the results of the protein and mRNA expression levels of PDCD4, VCAM-1 and ICAM-1 in coronary artery tissues of AS mice suggested that WWP2 decreases the endothelial injury in AS mice via the PDCD4/HO-1 pathway ( Supplementary Figures S3 and S4).

## Discussion

WWP2 is an E3 ubiquitin ligase of the HECT-type NEDD4 family, which plays important roles in various physiological activities through interacting with different substrates [32]. WWP2 is involved in the regulation of tumor progression by interacting with and degrading p27 and PTEN, in which WWP2 functions as a tumor-promoting factor [ 33– 35] . In addition, WWP2 serves as a positive regulator of osteogenesis and cartilage through RUNX2 ubiquitination and degradation [23]. Previous studies also demonstrated that WWP2 plays a crucial role in immune regulation and embryonic differentiation by regulating the ubiquitination of TRIF and Rpb1 [ 36, 37] . A recent study illustrated that WWP2 interacts with PARP1 and ubiquitinates its K249 and K418, which alleviates ISO-induced cardiac remodeling [24]. Furthermore, WWP2 plays key roles in VSMCs proliferation, migration, and phenotypic transformation, as well as hypertensive angiopathy, in which WWP2 shows a new non-ubiquitination function, i.e., regulating SIRT1-STAT3 acetylation and phosphorylation [25]. In this study, we found that injection of Ad-WWP2 significantly improved serum lipid levels and counteracted the endothelial injury in AS mice. Overexpression of WWP2 reduced ox-LDL-induced HUVECs oxidative stress and inflammation. Mechanistically, our study revealed that PDCD4 is a new substrate of WWP2. WWP2 overexpression increases PDCD4 ubiquitination level and promotes PDCD4 degradation, which is consistent with a study reporting that WWP2 ubiquitinates Septin4-K174 by interacting with the GTPase domain of Septin4, thereby reducing endothelial injury [26]. Although the present study is not intended to confirm the degradation of Septin4 mediated by WWP2, it can be predicted that WWP2 has a similar function in ox-LDL-stimulated endothelial cells at this time, that is, WWP2 inhibits endothelial cell oxidative stress and inflammation by mediating PDCD4 degradation. In addition, WWP2 also targets other important proteins with multiple functions, such as PTEN [ 21, 38] , PARP1 [24], ADAR2 [39] and Oct4 [40], for ubiquitin-mediated degradation. It will be interesting to determine whether these WWP2 targets are also involved in the endothelial injury mediated by oxidative stress.

As a tumor suppressor, PDCD4 has multi-functions including inhibiting cell growth, tumor invasion, metastasis, and inducing apoptosis [41]. PDCD4 was also demonstrated to play an important role in cardiovascular diseases such as hypertension, atherosclerosis, myocardial infarction, and ischemia-reperfusion injury. *PDCD4* knockout in mice potently blocks pulmonary caspase-3 activation and the development of chronic hypoxia plus SU 5416 PH, which reduces endothelial injury in pulmonary hypertension [42], which is consistent with the findings that silencing of *PDCD4* suppresses the apoptosis of human pulmonary artery smooth muscle cells [43]. It is indicated that PDCD4 may cause apoptosis-related cell injury. In this study, our results suggested that PDCD4 deficiency reduces the promoting effect of *WWP2* silencing on apoptosis in ox-LDL-stimulated HUVECs. Deficiency of PDCD4 significantly improves cardiac function, reduces infarcted tissue size, and prevents post-infarction-induced apoptosis in rats after MI [44]. PDCD4-mediated Akt signaling pathway results in vascular endothelial cell injury caused by lower-extremity ischemia-reperfusion in rats [45]. Increasing evidence supported that PDCD4 plays a negative role in the progression of atherosclerosis [ 12, 46] . The downregulation of PDCD4 expression was found to reduce the formation of atherosclerotic plaque by decreasing the accumulation of pro-inflammatory factors in ApoE ^–/–^ mice [17], which supported our results that PDCD4 deficiency decreased the concentrations of IL-1, IL-1β and TNF-α in ox-LDL-treated HUVECs.

It was reported that oxidative stress-induced cell injury is also related to the PDCD4 expression in cardiovascular diseases [47]. It has been confirmed that oxidant-induced endothelial injury, inflammatory response, foam cell formation in atherosclerotic plaque progression can be inhibited by nuclear factor erythroid 2-related factor 2 (Nrf2)/HO-1 activation [ 48, 49] . *HO-1* is a downstream gene of Nrf2, and increased expression of HO-1 is induced by the activation of Nrf2. As an inducible enzyme, HO-1 can increase NO level, decrease inflammatory factors levels, diminish atherosclerotic plaques, and inhibit the progression and destabilization of vulnerable plaques [ 50, 51] . HO-1 suppresses cell apoptosis by protecting against oxidative stress. It was found that HO-1 was reduced in patients with coronary atherosclerosis [52], which further suggested that HO-1 might be involved in the mechanism of AS. In this study, we demonstrated that *PDCD4* knockdown could weaken the inhibitory effect of ox-LDL treatment on OH-1 expression, and SnPP abolished the effects of *PDCD4* knockdown-induced inhibition of endothelial injury on ox-LDL-treated HUVECs. Our data showed that the alleviated-atherosclerosis induced by *PDCD4* knockdown is at least partly dependent on the increased expression of HO-1. Thus, all the above evidence revealed that PDCD4 plays an important role in modulating cell apoptosis via various pathways, including HO-1. Furthermore, it was reported that silencing of *WWP2* significantly suppressed Akt activation which was identified to trigger PDCD4 phosphorylation for ubiquitin binding [ 20, 53] . Therefore, we speculate that the Akt signaling may participate in WWP2 decrease/PDCD4/HO-1 axis to regulate endothelial injury in AS, which needs to be further investigated.

It was reported that abnormal expression of PDCD4 is regulated by many pathways including ubiquitination that is involved various diseases. Li *et al*. [54] reported that SCF SKP2 triggered K48-linked ubiquitination and degradation of PDCD4 in breast cancer. DTL interacts with PDCD4 and promotes the ubiquitin-proteasomal degradation of PDCD4, thus enhancing cancer progression [55]. PDCD4 is rapidly phosphorylated on Ser67 by the protein kinase S6K1 and subsequently ubiquitinated by the ubiquitin protein ligase beta-transducin repeat-containing protein (β-TrCP), followed by the degradation by the proteasome [20]. PDCD4 was degraded by the ubiquitin protein ligase β-TrCP in a phosphorylation-independent manner, which showed impaired myotube formation [56]. Moreover, Ge *et al.* [18] reported that pulsating shear stress could induce the β-TrCP-mediated degradation of PDCD4 in HUVECs, in which the reduction of PDCD4 may only participate in the proliferation of HUVECs, rather than in the process of apoptosis. Cell apoptosis-associated endothelial injury is the initial event and major cause of multiple cardiovascular diseases such as AS and hypertensive angiopathy. Therefore, the protective effect of PDCD4 reduction on endothelial injury in AS may partly depend on β-TrCP-mediated PDCD4 degradation. Previous reports [ 24, 26] and the present study demonstrate that WWP2 functions as a novel protective regulator against endothelial injury and vascular remodeling after oxidative stress-induced endothelial injury. Thus, the above evidence supports our results that WWP2-mediated PDCD4 ubiquitination may protect against endothelial injury in AS progression. However, whether the β-TrCP-mediated PDCD4 ubiquitination exhibits protective on endothelial injury in ox-LDL-stimulated HUVECs warrants further investigation. In this study, our findings suggest that WWP2 directly binds with PDCD4 and mediates PDCD4 degradation through the ubiquitin proteasome pathway. Here we addressed PDCD4 as another substrate of WWP2, thus participating the modulation of oxidative stress and inflammation in ox-LDL- stimulated induced HUVECs. However, the ubiquitination mechanism of WWP2 on PDCD4 in endothelial cells warrants further investigation.

In summary, this study reported the interaction between WWP2 and PDCD4. WWP2 overexpression increases PDCD4 ubiquitination level and degrades PDCD4. Functional experiments demonstrated that overexpression of WWP2 reduces ox-LDL-induced HUVECs oxidative stress and inflammation, as well as improves AS progression *in vivo* . Moreover, knockdown of *PDCD4* ameliorates the effects of *WWP2* silencing on ox-LDL-induced HUVECs injury. Thus, our findings suggest that WWP2 protects against AS progression via the ubiquitination of PDCD4.

## Supplementary Data

Supplementary Data is available at *Acta Biochimica et Biphysica Sinica* online.
